# X-box binding protein 1: A new metabolic mediator and drug target of metformin?

**DOI:** 10.3389/fphar.2022.1013218

**Published:** 2022-11-11

**Authors:** Kai Lou, Pei Sun, Chunxue Zhang, Qiang Jiang, Shuguang Pang

**Affiliations:** ^1^ Department of Endocrinology, Jinan Central Hospital, Shandong University, Jinan, China; ^2^ Department of Endocrinology, Jinan Central Hospital Affiliated to Shandong First Medical University, Jinan, China; ^3^ Department of Nuclear Medicine, Jinan Central Hospital, Jinan Central Hospital Affiliated to Shandong First Medical University, Jinan, China

**Keywords:** metformin, hypertriglyceridemia, diacylglycerol *O*-acyltransferase 2, X-box binding protein 1, AMP-activated protein kinase

## Abstract

Accumulating evidence has demonstrated that metformin improved hypertriglyceridemia. The present study aim to investigate the molecular mechanism by which metformin improves hypertriglyceridemia *via* regulation of diacylglycerol *O*-acyltransferase 2 (DGAT2) and X-box binding protein 1 (XBP1) in the liver and whether AMP-activated protein kinase (AMPK) is involved. Mice were fed a high-fat diet (HFD) or high-fat diet with metformin for 5 weeks to evaluate the effect of metformin on triglyceride (TG) levels and expression of DGAT2 and XBP1 in the liver. *In vitro* HepG2 cells or XBP1 knockout AML12 hepatocytes were stimulated with metformin, palmitic acid or small interfering RNA inducing XBP1 knockdown, or dominant-negative mutant AMPK plasmid. Metformin treatment reduced hepatic TG levels in the liver of HFD-fed mice. Expression of nuclear and cytoplasmic XBP1 protein and its downstream target gene DGAT2 decreased in the liver of HFD-fed mice and HepG2 cells after metformin treatment. AMPK inactivation or overexpression of XBP1 attenuates this effect. Our preliminary results demonstrate that metformin activates AMPK to reduce TG synthesis by inhibiting the XBP1-mediated DGAT2 pathway, at least in part, suggesting that XBP1 is a new metabolic mediator for metformin treatment of hypertriglyceridemia and associated metabolic disease.

## Introduction

Hypertriglyceridemia is becoming the most common form of hyperlipidemia, and increases the risks of acute pancreatitis, atherosclerotic cardiovascular disease (ASCVD), non-alcoholic fatty liver (NAFLD), and diabetes (Simha, 2020; Hernandez et al., 2021). The ensuing 60 years of research has indicated that there was a close correlation between hypertriglyceridemia and Type 2 diabetes mellitus (T2DM), which hypertriglyceridemia often existed years before the prediabetic and diabetic state (Berkowitz, 1966; Lorenzo et al., 2013; Li et al., 2020; Ye et al., 2022). Hypertriglyceridemia may contribute to an increased risk of T2DM by several possible mechanisms including impaired insulin sensitivity, decreased insulin secretion, impaired fatty acid oxidation, and muscle insulin resistance (Muoio and Newgard, 2008).

The prevalence of hypertriglyceridemia is 13.8% among Chinese adults, according to a nationally representative survey (Zhang et al., 2018). Treatment of hypertriglyceridemia could reduce the cardiovascular risk and improve the prognosis of metabolic diseases, while some traditional oral TG-lowering drugs, such as fenofibrate and nicotinic acid derivatives, have adverse effects and limit their clinical application (Miller et al., 2011; Berglund et al., 2012; Garber et al., 2019).

Metformin is recommended as the first choice for monotherapy for T2DM, (Garber et al., 2019), and metformin also exhibits protective benefits in lipid metabolism, tumorigenesis, and cardiovascular protection (Zhou et al., 2018). Clinical research has indicated that there were obvious clinical benefits of improved hypertriglyceridemia in diabetic and non-diabetic patients (Sato et al., 2019; Jiang et al., 2020; Zhao et al., 2021). The AMP-activated protein kinase (AMPK) signaling pathway is involved in the regulation of metformin-mediated lipid and glucose metabolism (Hawley et al., 2002; Zang et al., 2004). Previous studies demonstrated that AMPK decreases triglyceride (TG) and cholesterol synthesis by regulating acetyl-CoA carboxylase (Acc), sterol regulatory element-binding protein-1c (SREBP-1c), and stearyl-coenzyme A desaturase 1(Scd1) activity (Zang et al., 2004; Li et al., 2011; Zhu et al., 2018).

Diacylglycerol *O*-acyltransferase 2 (DGAT2) catalyzes the final and only committed step in the synthesis of TGs (Cases et al., 2001; Yen et al., 2008). Mice with hepatic overexpression of DGAT2T exhibit profound hepatic insulin resistance and steatosis (Jornayvaz et al., 2011). Inhibition of DGAT2 expression has been shown to improve insulin resistance and hepatic steatosis in T2DM rats, suggesting that DGAT2 plays an important role in hepatic steatosis and hypertriglyceridemia in T2DM (Choi et al., 2007; Zhang et al., 2019). X-box binding protein 1 (XBP1) functions as a transcription factor in response to endoplasmic reticulum (ER) stress, adipocyte differentiation, and fatty acid and TG synthesis, with *DGAT2* serving as a downstream target gene of XBP1 (Yoshida et al., 2001; Lee et al., 2008).

The underlying mechanism by which metformin improves hypertriglyceridemia remains to be elucidated. Therefore, the present study investigated whether metformin improves hypertriglyceridemia *via* regulation of DGAT2 or XBP1 in the liver and whether AMPK is involved. The results could facilitate the development of a new therapeutic agent for treating hypertriglyceridemia, NAFLD, and ASCVD with hypertriglyceridemia.

## Materials and methods

### Reagents and antibodies

Metformin hydrochloride was purchased from Sigma-Aldrich Co. (St. Louis, MO, United States). AMPK α1/α2 antibody and phospho-AMPK (Thr-172) antibody were obtained from Cell Signaling Technology (Beverly, MA, United States). DGAT2 polyclonal antibody (PA5-103785) was obtained from Invitrogen (Carlsbad, CA). Anti-XBP1antibody (ab37152) was purchased from Abcam (Cambridge, United Kingdom). GAPDH, β-tubulin, and β-actin antibodies were obtained from Proteintech (Chicago, IL, United States). BCA protein assay kits were obtained from Beyotime Biotechnology (Shanghai, China).

## Animals

All mice were kept in a specific pathogen–free room with a 12-h light/dark cycle, and the room temperature was maintained at 22 ± 2°C. The experimental procedures were approved by the Animal Care and Use Committee of Shandong First Medical University (GZR 2020-023-01) (Jinan, China). All protocols were performed according to the ethical guidelines for animal studies. At the end of the experiment, the mice were euthanized with intraperitoneal injection of sodium pentobarbital (40 mg/kg/body weight).

Seven-week-old male C57/BL6 mice were allowed to acclimatize to the environmental conditions for 1 week prior to the experiment and then randomly divided into three groups (*n* = 6–8 in each group): 1) control group receiving control diet (Research Diets D12450); 2) high-fat diet (Research Diet D12492, HFD-fed group) containing 60% of kcal from fat; and 3) HFD + metformin treatment group received a HFD with oral dose of metformin (300 mg/kg) as previously described. (Shih et al., 2014; Zabielski et al., 2018). The mice in each group were fed the respective diet for 5 weeks. The body weight and food intake of the mice were measured every week. Blood samples were collected to determine serum total cholesterol levels after the treatment period. At the end of the experiment, liver tissues were rapidly collected from all mice for simultaneous analysis, and the remnant livers were frozen in liquid nitrogen for subsequent protein analysis.

### Cell culture and treatment

Human hepatoma HepG2 cells were purchased from the Type Culture Collection of the Chinese Academy of Sciences (Shanghai, China) and cultured at 37°C and 5% CO_2_in complete Dulbecco’s modified Eagle’s media (DMEM) (Gibco, United States) containing 10% fetal bovine serum (Gibco), 1% penicillin/streptomycin (Invitrogen, United States), and 5.5 mmol/L d-glucose. XBP1 knockout AML12 hepatocyte was from Fenhui Biotechnologies Inc (Hunan, China) and cultured in DMEM/F12 media. (Zhu et al., 2018). For experiments, the HepG2 cells (∼80% confluence) were treated for 24 h with 2 mmol/L (mM) or 5 mM metformin in serum-free medium containing 30 mM glucose, 100 nM insulin, and0.25% bovine serum albumin. (Zang et al., 2004).

### Triglyceride assay

Liver TG content was measured using a glycerol-phosphate oxidase-peroxidase method and a TG assay kit (Applygen Technologies, Beijing, China) according to the manufacturer’s instructions.

### Western blotting

Total protein extraction and WB were conducted as described previously (Zang et al., 2004; Qi et al., 2018). Nuclear and cytoplasmic proteins from cells and mouse liver were extracted using a NE-PER nuclear and cytoplasmic extraction reagent kit (Pierce Biotechnology Inc.) according to the manufacturer’s instructions.

### Real-time quantitative PCR

Total RNA was extracted from mouse liver and cells using TRIzol reagent (Takara, Tokyo, Japan) and reverse-transcribed to cDNA using a Prime Script RT Reagent kit (Takara) according to the manufacturer’s instructions. cDNAs were analyzed by real-time quantitative PCR using SYBR Green Premix Ex Taq II (TaKaRa, Kusatsu, Japan) and a Roche 480 detection system. β-Actin was used as an internal reference. The relative gene expression levels were quantified according to the 2^−ΔΔCt^ method.

The PCR primer sequences are listed in Table 1.

**TABLE 1:** Quantitative RT-PCR primers.

Gene	Species	Forward primer	Reverse primer
*DGAT2*	mouse	TGG​CAT​TTG​ACT​GGA​ACA​CG	GGA​AAG​TAG​TCT​CGG​AAG​TAG​CG Ma et al. (2015)
*XBP1*	mouse	ATT​CTG​AGT​CTG​ATA​TCC​TTT​TGG​G	TCC​AGC​TTG​GCT​GAT​GAG​GT
*β-actin*	mouse	ACC​CCA​GCC​ATG​TAC​GTA​GC	GTG​TGG​GTG​ACC​CCG​TCT​C
*Acc1*	mouse	CGC​CAA​CAA​TGG​TAT​TGC​AGC	TCG​GAT​TGC​ACG​TTC​ATT​TCG
*Fas*	mouse	GGA​GGT​GGT​GAT​AGC​CGG​TAT	TGG​GTA​ATC​CAT​AGA​GCC​CAG
*Scd1*	mouse	TTC​TTG​CGA​TAC​ACT​CTG​GTG​C	CGG​GAT​TGA​ATG​TTC​TTG​TCG​T
*Gpat1*	mouse	CTT​GGC​CGA​TGT​AAA​CAC​ACC	CTT​CCG​GCT​CAT​AAG​GCT​CTC
*DGAT2*	human	TGG​GGG​CTG​GTG​CCC​TAC​TC	AAT​TGG​CCC​CGA​AGG​CTG​GC Zhang et al. (2019)
*XBP1*	human	ATG​GAT​TCT​GGC​GGT​ATT​GAC​T	AGA​GAA​AGG​GAG​GCT​GGT​AAG​G
*β-actin*	human	ACA​GAG​CCT​CGC​CTT​TGC​CG	ACA​TGC​CGG​AGC​CGT​TGT​CG

### Plasmid or adenoviruses transfection of cells

The plasmids encoding the dominant-negative mutant AMPK (DN-AMPK).

Have been previously described. (Ma et al., 2015). The empty vector pc-DNA3 was used as a control. The recombinant adenoviruses expressing XBP1were obtained from Hanbio Biotechnology Co. (Shanghai, China).

For transient expression assays, HepG2 cells were cultured in complete medium with 10% FBS to 80% confluence, synchronized overnight in serum-free DMEM in 35-mm plates, and then transfected with plasmids or adenoviruses in the presence or absence of metformin for 48 h using Lipofectamine 3000 (Invitrogen, Carlsbad, CA) in accordance with the manufacturer’s protocols. After 48 h, the cells were harvested and utilized for subsequent analyses.

### RNA interference

Small interfering RNAs targeting XBP1 (XBP1 siRNA) and a scrambled siRNA (control siRNA) were synthesized by Gene Pharma Corporation (Shanghai, China).

HepG2 cells were transfected with siRNAs using Lipofectamine 3000 by following the manufacturer’s instructions. HepG2cells were transfected with XBP1 siRNA, scrambled siRNA, or stimulated with metformin in 35-mm plates with a corresponding volume of transfection reagent. After 48 h, the cells were harvested for further protein and mRNA analyses.

### Luciferase reporter assay

The DGAT2 promoter (−1,000 to −1) luciferase plasmid was obtained from GeneChem Corporation (Shanghai, China). HepG2 cells were co-transfected with 1 μg of the promoter-luciferase reporter plasmids and XBP1 siRNA for 48 h or stimulated with metformin. Renilla luciferase plasmid pRL-SV40 (Promega, Madison, WI, United States) was used as an internal control. Firefly luciferase activity was normalized to Renilla luciferase activity using a dual-luciferase reporter assay system (Promega) (Ma et al., 2015).

### Immunofluorescence staining

Immunofluorescence staining was performed as described previously (Li et al., 2017). Briefly, HepG2 cells were fixed with 4% paraformaldehyde for 15 min and permeabilized in phosphate-buffered saline containing 0.15% Triton X-100 for 5 min. After blocking with 10% normal mouse serum for 30 min, the cover slides were incubated with the appropriate primary antibody (1:100) at 4°C overnight and subsequently with TRITC-conjugated secondary antibody. Cell nuclei were stained with 4′-6-diamidino-2-phenylindole (DAPI) (Vector Laboratories, Burlingame, CA, United States). Specimens were visualized using an Olympus BX53 microscope (Olympus, Japan).

### Statistical analysis

The data are expressed as the mean ± standard error of the mean (SEM). All experiments were repeated at least three independent times. Differences of intergroup comparisons were analyzed using one-way analysis of variance followed by Tukey’s multiple comparison tests using Prism (version 5; GraphPad Software Inc., San Diego, CA, United States). Differences between groups were considered significant at *p* < 0.05.

## Results

### Metformin treatment reduced hepatic TG content in mice

**FIGURE 1:**
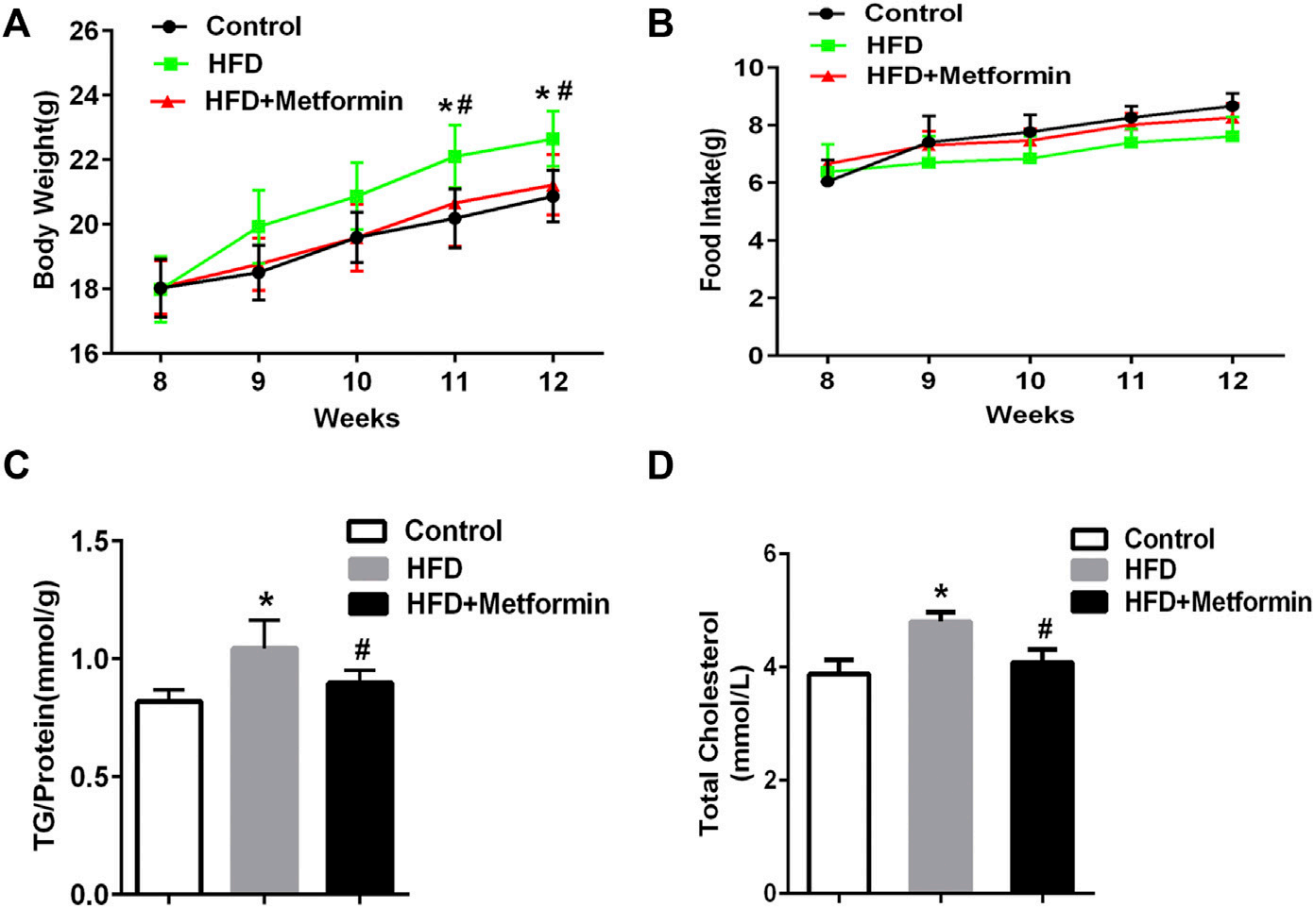
Metformin treatment reduced hepatic TG content in mice. **(A)** Body weight, **(B)** total food intake, **(C)** cellular TG content in the liver, and **(D)** total serum cholesterol in mice fed control diet or HFD with or without metformin treatment for 5 weeks. Data are presented as the mean ± SEM. ∗p < 0.05 compared with control mice. #p < 0.05 compared with HFD-fed mice, *n* = 6–8.

To determine if metformin affects the cellular TG content in the liver, mice were fed a HFD or HFD with metformin for 5 weeks. A significant body weight gain was observed in HFD-fed mice after 4 weeks compared with mice in the control group, but metformin-treated mice lost weight (Figure 1A). There was no significant difference in food take between the control, HFD-fed, and HFD with metformin groups (Figure 1B).

The cellular TG content in the liver was increased in HFD-fed mice, but metformin treatment exhibited an inhibitory effect on TG accumulation in the liver of HFD-fed mice (Figure 1C). Total serum cholesterol levels were also reduced in HFD-fed mice after metformin treatment (Figure 1D). These results suggest that metformin reduces the hepatic TG content and total serum cholesterol in mice.

### Metformin decreased DGAT2 expression in the liver of mice and in HepG2 cells

**FIGURE 2:**
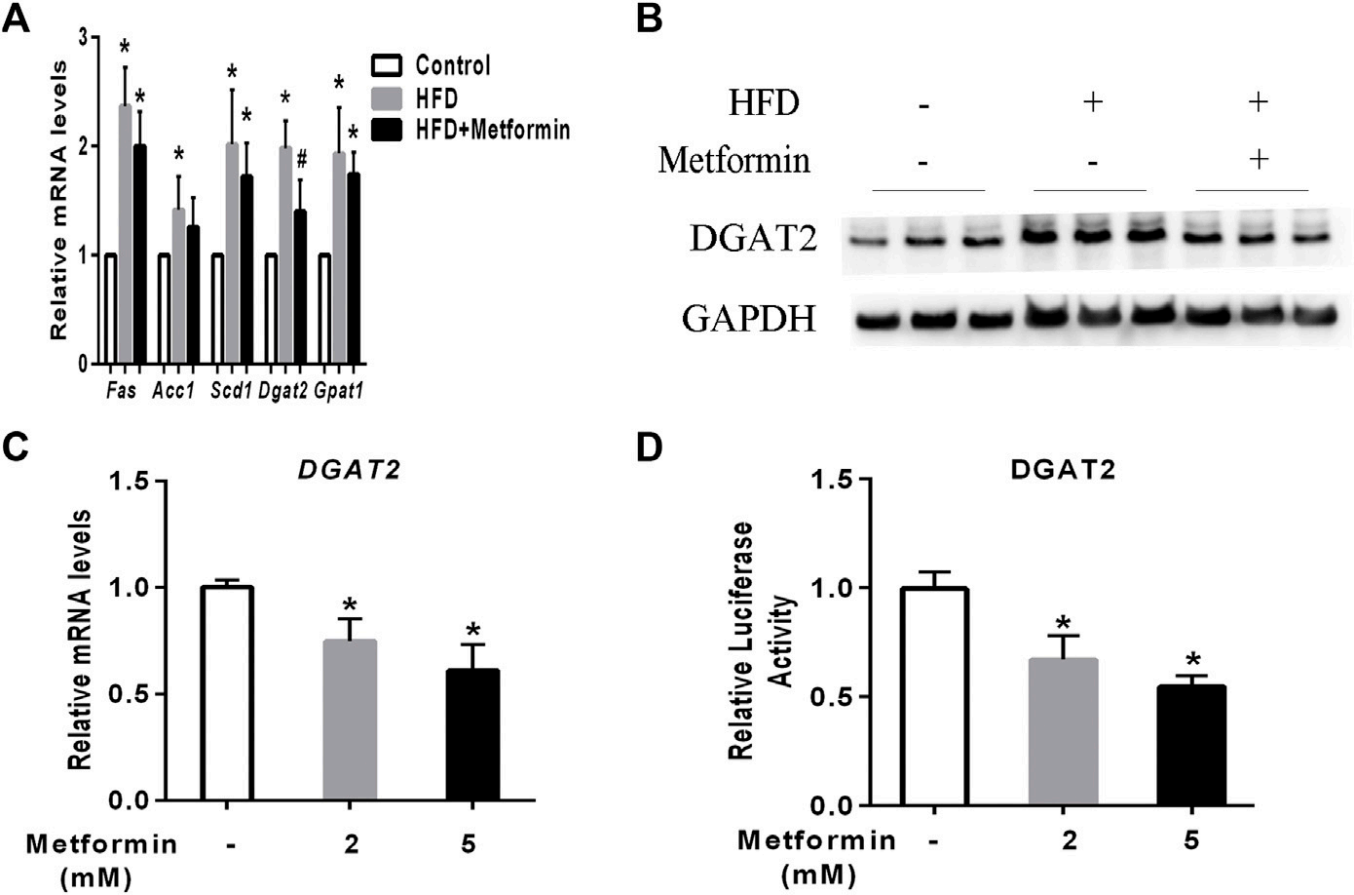
Metformin decreased DGAT2 expression in the liver of mice and in HepG2 cells. **(A)** The hepatic mRNA expression of lipogenic genes and **(B)** DGAT2 protein in the liver of the mice fed control diet and HFD with or without metformin treatment for 5 weeks. ∗p < 0.05 compared with control mice. #p < 0.05 compared with HFD-fed mice, *n* = 6–8. **(C)** Relative levels of DGAT2 mRNA in HepG2 cells treated with 2 mmol/L or 5 mmol/L metformin for 24 h. **(D)** Relative luciferase activity of DGAT2 in HepG2 cells treated with 2 mmol/L or 5 mmol/L metformin for 24 h ∗p < 0.05 compared with control. Data are presented as the mean ± SEM of at least three independent experiments.

To investigate the main mediator of metformin on TG biosynthesis, The key hepatic mRNA expression of lipogenic genes such as fatty acid synthetase (Fas), stearyl-coe nzyme A desaturase 1(Scd1), acetyl coA carboxylase 1 (Acc1), glycerol-3-phosphate acyltransferase 1 (Gpat1), and DGAT2 were detected. (Alves-Bezerra and Cohen, 2017). HFD-fed induced a significant increase of lipogenic genes expression including Fas, Scd1, Acc1, Dgat2, and Gpat1 in mice, metformin treatment led to a slight decrease of Fas, Scd1, Acc1, and Gpat1expression, while the mRNA of DGAT2 represented a significant decrease in the liver of metformin-treated mice compared to the HFD-fed mice (Figure 2A). Further, western blotting analysis demonstrated that the protein level of DGAT2 were also changed in accordance with its mRNA expression level in mice after metformin treatment (Figure 2B). To confirm this findings, HepG2 cells were stimulated with different concentrations of metformin, which resulted in a dose-dependent significant decrease in the mRNA level and luciferase activity of DGAT2 (Figures 2C,D). These data indicate that DGAT2 is involved in the metformin-induced inhibition of TG synthesis.

### Metformin decreased nuclear and cytoplasmic XBP1 protein levels in the liver

**FIGURE 3:**
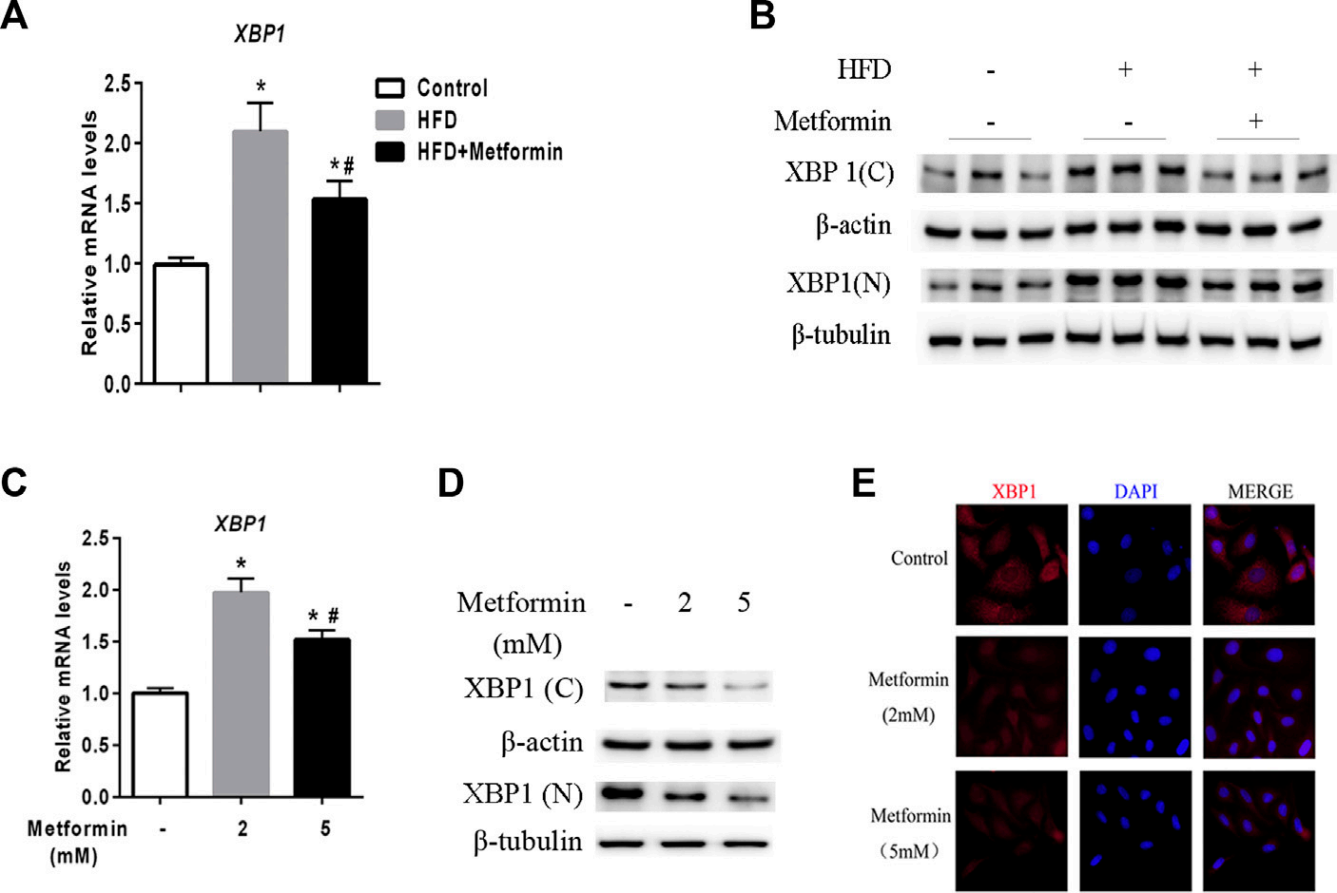
Metformin decreased nuclear and cytoplasmic XBP1 levels in the liver. **(A)** XBP1 gene expression in the liver of the mice fed control diet or HFD with or without metformin treatment for 5 weeks. **(B)** Nuclear (N) and cytoplasmic **(C)** XBP1 protein in the liver of the mice fed by control diet or HFD with or without metformin treatment for 5 weeks. ∗p < 0.05 compared with control mice. #p < 0.05 compared with HFD mice, *n* = 6–8. **(C)** Relative mRNA level, **(D)** Nuclear (N) and cytoplasmic **(C)** XBP1 protein levels, and **(E)** immunofluorescence images of XBP1 (red) and nuclear staining with 4′,6-diamidino-2-phenylindole (DAPI, blue) in HepG2 cells treated with 2 mmol/L or 5 mmol/L metformin for 24 h. Magnification, ×200. Data are presented as the mean ± SEM of at least three independent experiments. ∗p < 0.05 compared with control cells. #p < 0.05 compared with 2 mmol/L metformin-treated cells.

A prior study demonstrated that XBP1 is an upstream regulator of DGAT2 (Lee et al., 2008). Two forms of XBP1 have been identified unspliced form (XBP-1u) and spliced form (XBP-1s), XBP-1u undergo a unconventional cytoplasmic splicing processing under stress conditions to converse a XBP-1s form, which translocates into nucleus as an active transcription factor (Ron and Walter, 2007; Yanagitani et al., 2009; Sekimoto and Yoneda, 2012; Wu et al., 2015). Using an anti-XBP1 antibody that recognizes both forms of XBP1, we analyzed the expression of nuclear (N) and cytoplasmic (C) XBP1 protein in the liver of mice and HepG2 cells after metformin treatment. As shown in Figure 3A, XBP1 expression was increased in the liver of HFD-fed mice, but metformin treatment reduced the XBP1 mRNA level. The levels of both the nuclear and cytoplasmic forms of XBP1 protein were also significantly increased in the liver of HFD-fed mice, but metformin treatment alleviated this effect (Figure 3B).

Consistent with the *in vivo* data, XBP1 expression exhibited a clear dose-dependent decrease in HepG2 cells stimulated with metformin *in vitro* (Figures 3C–E). These results demonstrate that metformin inhibits XBP1 expression, thus playing an important role in the regulation of DGAT2 (Lee et al., 2008).

### Metformin decreased TG synthesis through regulating XBP1-mediated DGAT2 expression

**FIGURE 4:**
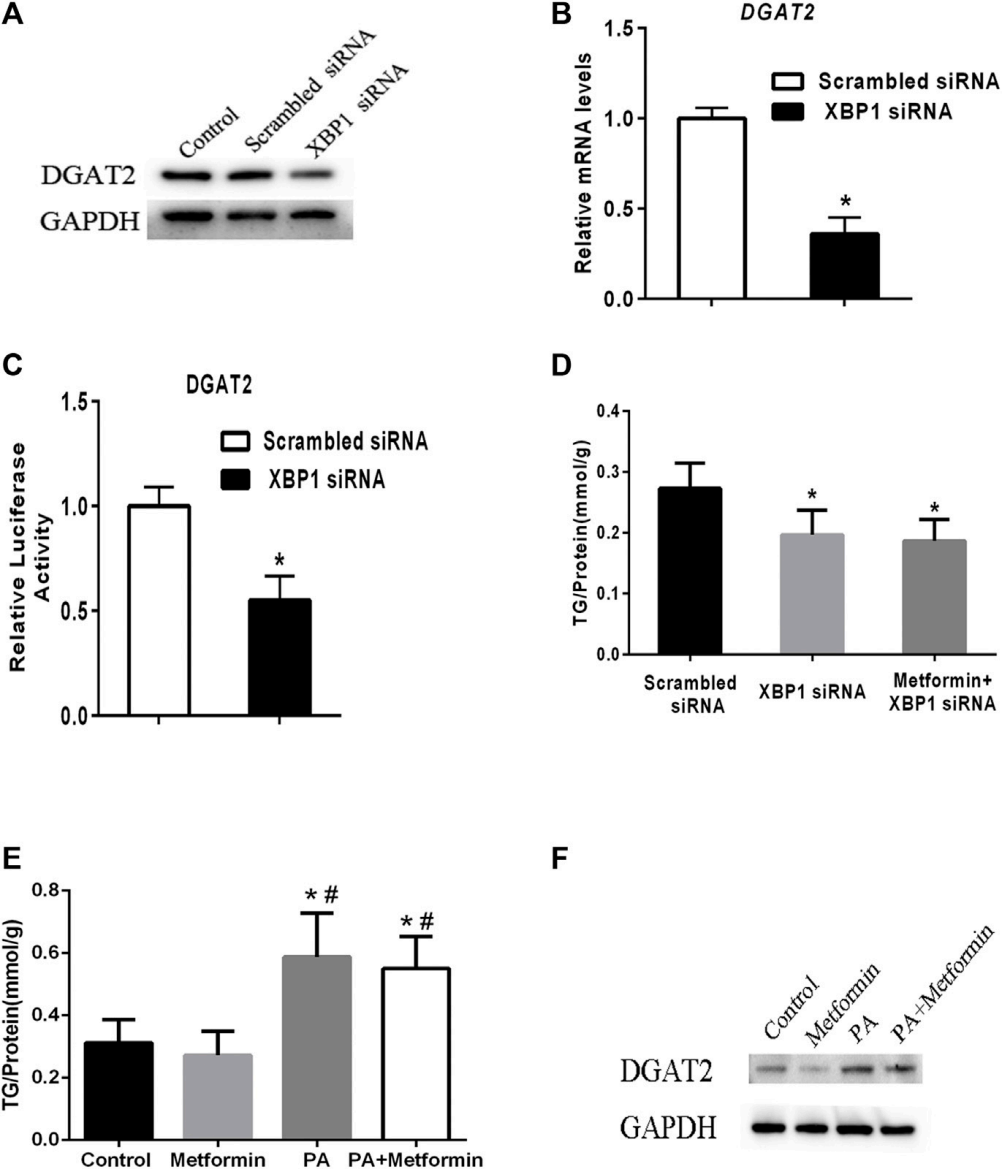
Metformin decreased TG synthesis by regulating XBP1-mediated DGAT2 expression. **(A)** HepG2 cells were transfected with small interfering RNAs targeting XBP1 (XBP1 siRNA) or a scrambled siRNA (control siRNA) or vehicle control for 48 h. DGAT2 proteins levels were analyzed by western blotting. **(B)** Relative mRNA level and **(C)** relative luciferase activity of DGAT2 in HepG2 cells transfected with XBP1 siRNA or scrambled siRNA for 48 h ∗p < 0.05 compared with scrambled siRNA. **(D)** TG content in HepG2 cells transfected with scrambled siRNA or XBP1 siRNA with or without metformin for 48 h ∗p < 0.05 compared with scrambled siRNA. **(E)** The intracellular TG contents were measured in XBP1 knockout AML12 hepatocytes treated with 2 mmol/L metformin with or without 200 μM palmitic acid (PA) for 48 h. **(F)** The DGAT2 proteins were determined. ∗p < 0.05 compared with control cells. #p < 0.05 compared with metformin-treated cells. Data are presented as the mean ± SEM. All experiments were conducted in duplicate.

To test the regulatory effect of XBP1 on DGAT2 expression, XBP1 siRNA was transfected into HepG2 cells. Levels of DGAT2 protein and mRNA were decreased compared with scrambled siRNA transfectants or control cells (Figures 4A,B). DGAT2 luciferase reporter assay results supported the above results (Figure 4C). There was no significant difference in hepatic TG content between XBP1 siRNA–treated HepG2 cells and cells treated with metformin and XBP1 siRNA (Figure 4D), suggesting that metformin-induced effects on TG synthesis are associated with XBP1.

To further explore the role of XBP1 in metformin-induced effect on DGAT2 and TG, XBP1 knockout AML12 hepatocytes were treated with 2 mmol/L metformin for 48 h or 200 μM palmitic acid (PA) with or without metformin for 48 h. The TG content was significant increased after PA incubation, but metformin treatment did not have an effect on TG content in XBP1 knockout AML12 hepatocytes after PA incubation suggesting that XBP1 play an important role in metformin-induced decrease of TG synthesis (Figure 4E). In addition, DGAT2 protein was significant increased in XBP1 knockout AML12 hepatocytes when treated by PA, metformin treatment exerted a mild inhibitory effect on DGAT2, but did not completely inhibit the PA-induced increase of DGAT2 protein suggesting that other mediators were involved in regulation of DGAT2 expression except XBP1 (Figure 4F). Taken together, these data reveal that metformin decreases TG synthesis by regulating XBP1-mediated DGAT2 expression, at least partly.

### The effect of metformin on TG synthesis *via* the XBP1-mediated DGAT2 pathway involved AMPK

**FIGURE 5:**
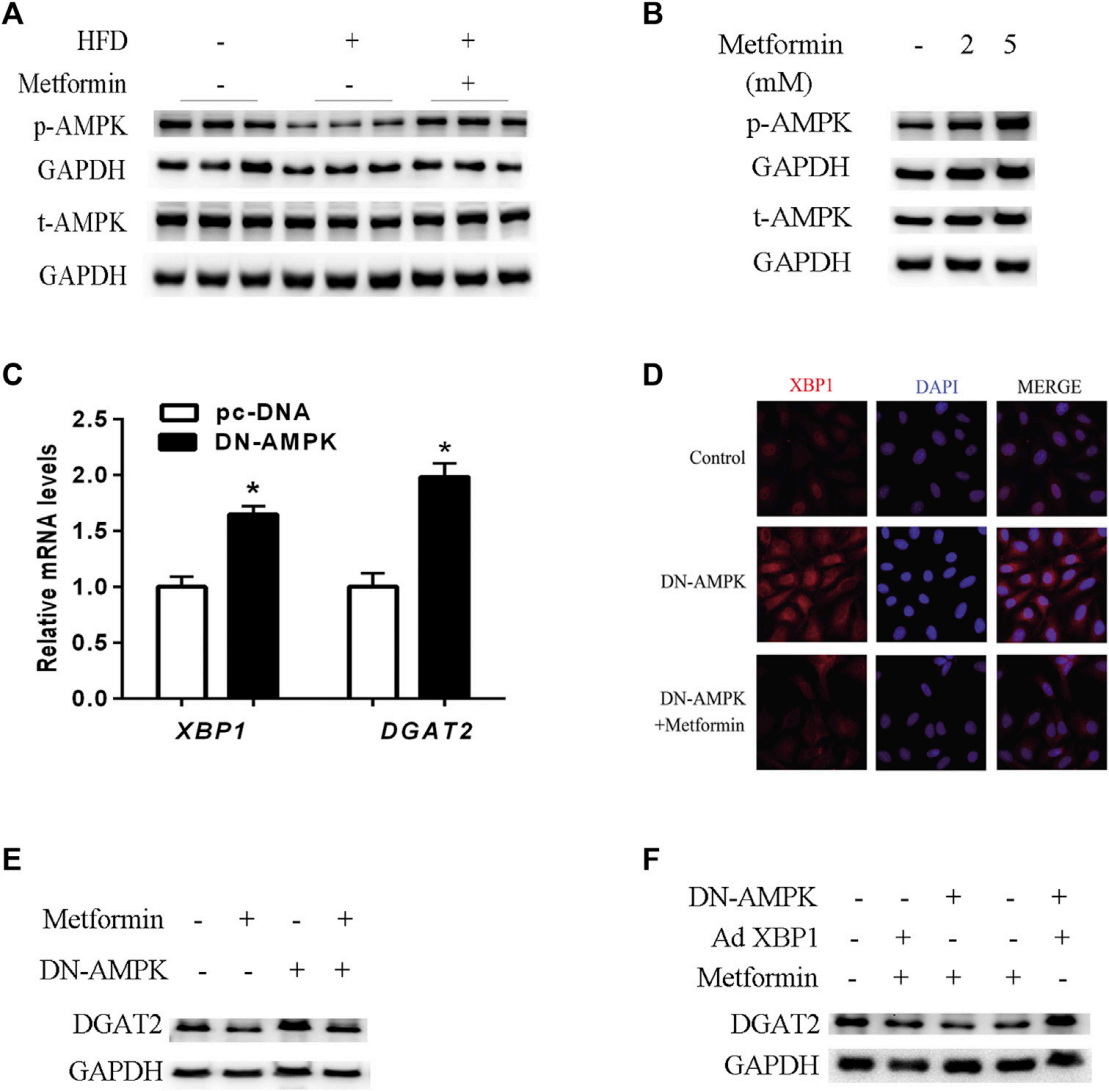
AMPK regulated the effect of metformin on TG synthesis via the XBP1-mediated DGAT2 pathway. **(A)** p-AMPK (Thr172) and total AMPK protein (t-AMPK) levels of mice fed control diet, HFD, or HFD with or without metformin treatment for 5 weeks; *n* = 6–8. **(B)** p-AMPK and t-AMPK levels in HepG2 cells treated with 2 mmol/L or 5 mmol/L metformin for 24 h. **(C)** Relative levels of XBP1 and DGAT2 mRNA in HepG2 cells transfected with dominant negative (DN)-AMPKα1 plasmid or pc-DNA empty vector for 48 h. Data are presented as the mean ± SEM. ∗p < 0.05 compared with control cell, respectively. **(D)** Immunofluorescence images of XBP1 and DGAT2 protein in HepG2 cells transfected with DN-AMPKα1 plasmid in the presence or absence of metformin for 48 h **(E)** DGAT2 protein levels in HepG2 cells transfected with DN-AMPKα1plasmid in the presence or absence of metformin for 48 h **(F)** DGAT2 protein levels in HepG2 cells co-transfected with DN-AMPKα1 plasmid or recombinant adenovirus expressing XBP1 (Ad XBP1) in the presence or absence of metformin for 48 h. These experiments were repeated at least three times. Representative blots of independent experiments are shown. GAPDH was used as a loading control.

The AMPK signaling pathway plays a key role in the molecular mechanisms of metformin’s multiple physiological functions. (Kim et al., 2007; Hardie, 2014; Song et al., 2015; Zhou et al., 2018). As shown in Figures 5A,B, metformin increased phosphorylated-AMPK (p-AMPK) levels and stimulated AMPK activity in mice and HepG2 cells. To verify the effect of AMPK activity on XBP1 and DGAT2 expression, HepG2 cells were transfected with DN-AMPKα1 plasmid, and AMPK activity was inhibited for 48 h *in vitro*. Inhibition of AMPK activity led to upregulation of XBP1 and DGAT2 expression in HepG2 cells (Figure 5C). Similar to the results shown in Figure5D, immunofluorescence staining clearly illustrated that XBP1 and DGAT2 proteins were enhanced by DN-AMPK, and metformin attenuated this change. DGAT2 protein levels were also increased by inhibition of AMPK activity, and this effect was also suppressed by metformin treatment (Figure 5E).

In order to further determine whether AMPK activity was involved in the metformin-induced regulation of the XBP1/DGAT2 pathway, DN-AMPK plasmid and recombinant adenoviruses expressing XBP1 (Ad XBP1) were co-transfected into HepG2 cells. Overexpression of XBP1 and inactivation of AMPK significantly increased DGAT2 protein levels compared with Ad XBP1 plus metformin (Figure 5F). Hence, we provide *in vivo* and *in vitro* evidence that AMPK activity plays a role in the effect of metformin in regulating the XBP1/DGAT2 pathway.

## Discussion

Accumulating evidence has demonstrated that metformin as a first-line oral antidiabetic drug is safe and well-tolerated and exhibits other potential beneficial effects on lipid metabolism and cancer (Song et al., 2015; Sato et al., 2017; Malinska et al., 2018; Zhou et al., 2018; Zhu et al., 2018). The current study showed that hepatic TG was significantly elevated in liver of HFD-fed mice; however, metformin alleviated hepatic TG accumulation, accompanied by an improvement in serum cholesterol. These findings were in accordance with earlier studies suggesting a beneficial lipid-lowering effect of metformin (Song et al., 2015; Choi et al., 2017; Zhu et al., 2018). In contrast, a recent report showed that metformin treatment improved weight loss and insulin resistance in humans without improvement in intrahepatic TG content, partly through increased hepatic fatty acids (FA) synthesis (Green et al., 2022). The discrepancy in findings with our results may due to dose and duration of metformin exposure or differential experimental condition such as diet structure.

DGAT2 is a key enzyme responsible for TG biosynthesis (Yen et al., 2008). DGAT2 overexpression in the liver of mice increases hepatic TG content, leading to NAFLD and hepatic insulin resistance (Jornayvaz et al., 2011). An early study reported that Tangduqing granules decrease DGAT2 expression, resulting in a relief of insulin resistance and hypertriglyceridemia in a T2DM rat model (Zhang et al., 2019). A previous study has demonstrated that metformin could reduce DGAT2 expression and hepatic lipid biosynthesis in diabetic mice, but the underlying molecular mechanism was unclear (Choi et al., 2017). In consistence with previous study, our observation indicated that metformin also decreased hepatic DGAT2 expression both *in vivo* and *in vitro*. Thus, DGAT2 may play a role in the metformin-induced TG-lowering effect. However, the mechanism by which metformin inhibits DGAT2 expression remains unknown.

A prior study reported that XBP1 is an upstream transcription factor of DGAT2 (Lee et al., 2008). XBP1 protein is present in unspliced and spliced forms in the cytoplasm and nucleus, respectively, and spliced XBP1 (active form) translocates to the nucleus to activate the transcription of target genes (Ron and Walter, 2007; Sha et al., 2009; Lee et al., 2011; Yu et al., 2018). Hence we focused on the effect of metformin on the nuclear form of XBP1 in the present study. Our data showed that HFD induces the increase in both nuclear and cytoplasmic XBP1 protein levels in line with Song et al. reported, (Yu et al., 2018), and XBP1 in turn significantly upregulates DGAT2 expression, whereas metformin suppresses both nuclear and cytoplasmic XBP1 levels and reduces XBP1-mediated DGAT2 expression *in vivo* in mice and in hepG2 cells. Moreover, siRNA-mediated konckdown of XBP1 and XBP1 knockout attenuated the effect of metformin-induced lowering of TG levels, though XBP1 knockout could not completely inhibit expression of DGAT2 suggesting that XBP1 is involved in the effect of metformin on TG synthesis and DGAT2, at least partly. Previous studies demonstrated that XBP1 is closely associated with ER stress and formation of atherosclerotic lesions involving apoptosis (Liu et al., 2009; Zeng et al., 2009). These preliminary findings suggest that XBP1 is likely a mediator of metformin’s multiple effects on the regulation of lipid metabolism, oxidative stress, and autophagy (Yoshida et al., 2001; Lee et al., 2008; Zhao et al., 2013; Ren et al., 2020). However, the molecular mechanism by which metformin inhibits XBP1 expression remains unknown.

AMPK is a regulator of energy homeostasis, and a growing number of studies indicate that the AMPK signaling pathway is involved in the effects of metformin (Foretz et al., 2014; Vasamsetti et al., 2015; Rena et al., 2017; Van Nostrand et al., 2020). Consistent with prior observations, metformin activates AMPK to decrease TG synthesis in the liver (Song et al., 2015) and inhibit XBP1-mediated DGAT2 expression, which is attenuated by AMPK inactivation. Further research is needed to explore the possible mechanism by which AMPK suppresses XBP1 activity, such as *via* phosphorylation modification or transcriptional regulation.

Our preliminary study has strengths and limitations. A notable strength is that we firstly uncover the linkage between XBP1and metformin, which maybe broaden our horizon about XBP1-mediated metabolic effect in liver. However, our results are subject to certain limitations. Metformin was involved in lipogenesis through regulating many lipogenic genes or regulatory enzyme such as forkhead box O1, SREBP-1c, Acc, Scd1, and Fas (Zang et al., 2004; Li et al., 2011; Zhao et al., 2013; Tyszka-Czochara et al., 2017). Besides, XBP1 was also implicated in SREBP-1c-associated lipogenic pathway (Ning et al., 2011; Yu et al., 2018). However, our experiment cannot completely exclude the influence of these mixed factors. For example, in support of the potential modulatory actions of metformin on XBP1, some more convincing evidence is needed from mice bearing a hepatocyte-specific deletion of XBP1. Furthermore, XBP1 functioned as a stress-inducible protein in unfolded protein response signaling under oxidative stress, we did not evaluate whether XBP1-mediated oxidative stress responses have a impact on metformin effect on lipogenesis in our study (Liu et al., 2009; Ning et al., 2011). In spite of these limitations, the study broadens our view that XBP-1 is at least partially involved in metformin function.

## Conclusion

**FIGURE 6:**
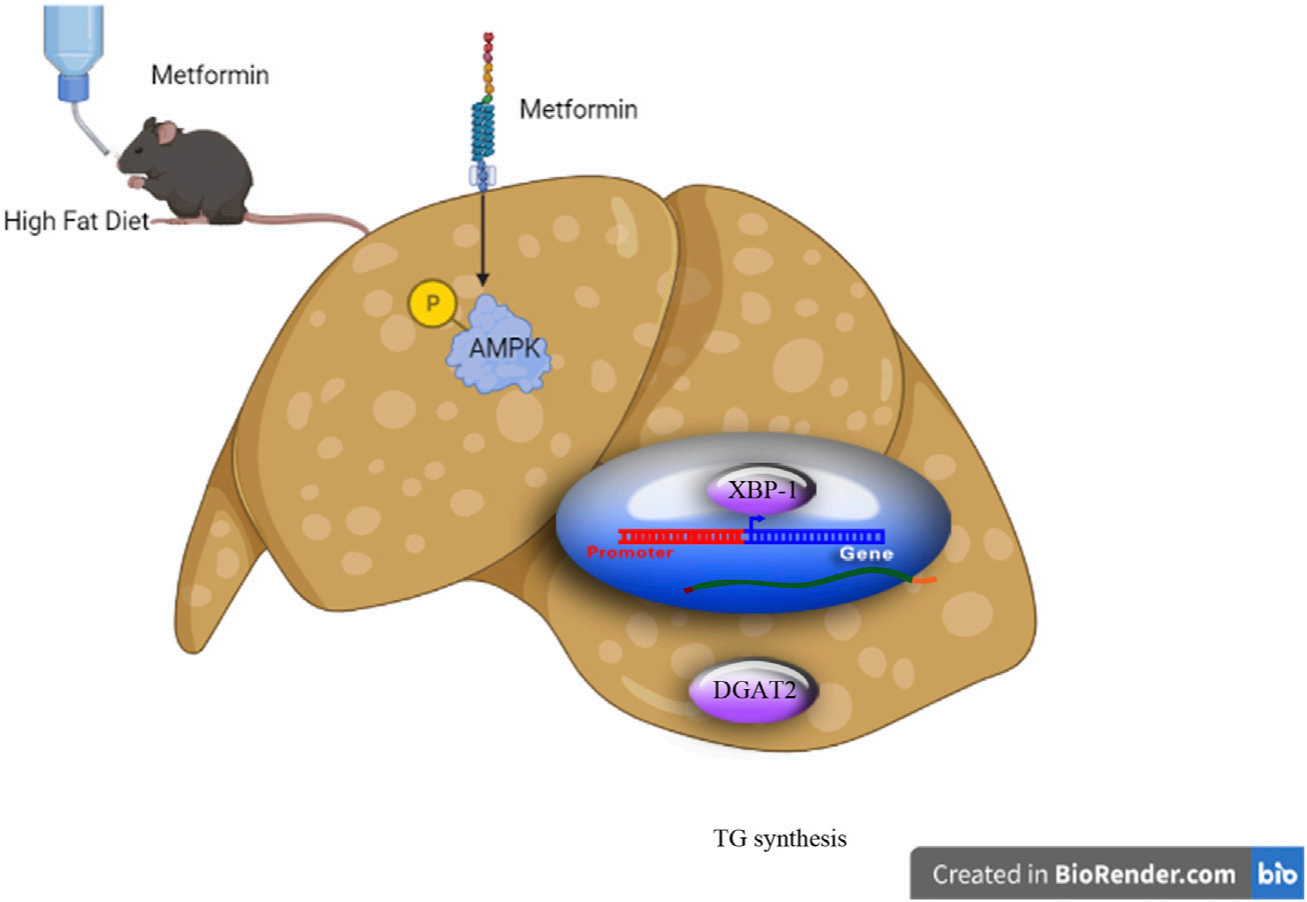
Schematic diagram of the experimental protocol and proposed mechanism for the triglyceride-lowering effect of metformin. (Schematic diagram created using Biorender.com).

Our preliminary results demonstrate that metformin activates AMPK to reduce TG synthesis by inhibiting the XBP1-mediated DGAT2 pathway, at least in part, suggesting that XBP1 is a new metabolic mediator for metformin treatment of hypertriglyceridemia and associated metabolic diseases (Figure 6).

## Data Availability

The original contributions presented in the study are included in the article/Supplementary Material, further inquiries can be directed to the corresponding author.
